# Genomic and transcriptomic correlates of immunotherapy response within the tumor microenvironment of leptomeningeal metastases

**DOI:** 10.1038/s41467-021-25860-5

**Published:** 2021-10-12

**Authors:** Sanjay M. Prakadan, Christopher A. Alvarez-Breckenridge, Samuel C. Markson, Albert E. Kim, Robert H. Klein, Naema Nayyar, Andrew W. Navia, Benjamin M. Kuter, Kellie E. Kolb, Ivanna Bihun, Joana L. Mora, Mia Solana Bertalan, Brian Shaw, Michael White, Alexander Kaplan, Jackson H. Stocking, Marc H. Wadsworth, Eudocia Q. Lee, Ugonma Chukwueke, Nancy Wang, Megha Subramanian, Denisse Rotem, Daniel P. Cahill, Viktor A. Adalsteinsson, Jeffrey W. Miller, Ryan J. Sullivan, Scott L. Carter, Priscilla K. Brastianos, Alex K. Shalek

**Affiliations:** 1 Department of Chemistry, Massachusetts Institute of Technology, Cambridge, MA USA; 2 Institute for Medical Engineering & Science, Massachusetts Institute of Technology, Cambridge, MA USA; 3 Broad Institute, Harvard University & Massachusetts Institute of Technology, Cambridge, MA USA; 4 Koch Institute for Integrative Cancer Research, Massachusetts Institute of Technology, Cambridge, MA USA; 5 Ragon Institute, Harvard University, Massachusetts Institute of Technology, & Massachusetts General Hospital, Cambridge, MA USA; 6 Department of Neurosurgery, Harvard Medical School & Massachusetts General Hospital, Boston, MA USA; 7 Division of Computational Biology, Dana-Farber Cancer Institute, Boston, MA USA; 8 Department of Medicine, Harvard Medical School & Massachusetts General Hospital, Boston, MA USA; 9 Massachusetts General Hospital Cancer Center, Boston, MA USA; 10 Division of Neuro-Oncology, Dana-Farber Cancer Institute, Boston, MA USA; 11 Department of Biostatistics, Harvard TH Chan School of Public Health, Boston, MA USA; 12 Division of Health Science & Technology, Harvard Medical School, Cambridge, MA USA; 13 Program in Computational & Systems Biology, Massachusetts Institute of Technology, Cambridge, MA USA

**Keywords:** Genomics, Cancer immunotherapy, CNS cancer, Metastasis

## Abstract

Leptomeningeal disease (LMD) is a devastating complication of solid tumor malignancies, with dire prognosis and no effective systemic treatment options. Over the past decade, the incidence of LMD has steadily increased due to therapeutics that have extended the survival of cancer patients, highlighting the need for new interventions. To examine the efficacy of immune checkpoint inhibitors (ICI) in patients with LMD, we completed two phase II clinical trials. Here, we investigate the cellular and molecular features underpinning observed patient trajectories in these trials by applying single-cell RNA and cell-free DNA profiling to longitudinal cerebrospinal fluid (CSF) draws from enrolled patients. We recover immune and malignant cell types in the CSF, characterize cell behavior changes following ICI, and identify genomic features associated with relevant clinical phenomena. Overall, our study describes the liquid LMD tumor microenvironment prior to and following ICI treatment and demonstrates clinical utility of cell-free and single-cell genomic measurements for LMD research.

## Abstract (web summary)

Leptomeningeal disease (LMD) is a serious complication of metastatic solid tumors with a poor prognosis. Here, by using single-cell RNA sequencing of cerebrospinal fluid, the authors report genomic and immune correlates of response to immunotherapy in two cohorts of patients with LMD treated with immune checkpoint inhibitors.

## Introduction

LMD—the infiltration of tumor cells into the leptomeninges and CSF—is an especially devastating complication of solid tumor malignancies, as it is usually rapidly fatal, with a median survival of about 4–6 weeks^1^. Approximately 5–8% of all cancer patients develop LMD^1–3^, with common histologies including breast cancer, lung cancer, and melanoma^2,3^. Furthermore, over the past decade, the incidence of LMD has risen due to increased patient survival through better tolerated and more effective treatment strategies. An effective systemic therapy for LMD is thus urgently needed, as current measures (e.g., craniospinal radiation and intrathecal therapies) have uncertain benefit and significant adverse effects^4^.

Immune checkpoint inhibitors (ICI) have revolutionized the field of oncology, and demonstrated remarkable response rates in a variety of metastatic, chemotherapy-refractory solid tumors^5–7^. More recently, ICI has emerged as a promising option for central nervous system (CNS) metastases. Preclinical data have demonstrated infiltration of T cells and programmed death-ligand 1 (PD-L1) expression in brain metastases (BM) of various histologies^8,9^, suggesting the potential for ICI to be efficacious in the CNS. Relatedly, ICI for metastatic melanoma and non-small cell lung cancer parenchymal brain metastases has demonstrated objective intracranial responses at a rate similar to systemic disease^10,11^. To our knowledge, however, ICI as treatment for LMD has not been evaluated in prospective clinical trials.

To address this unmet need, we initiated two phase II clinical trials of ICI in patients with LMD of any histology (NCT02886585, NCT02939300; see Methods). One trial (NCT02886585) evaluated the efficacy of an antibody targeting programmed cell death protein 1 (PD-1, drug name pembrolizumab) and the other (NCT02939300) evaluated targeting of PD-1 (drug name nivolumab) and cytotoxic T-lymphocyte-associated protein 4 (CTLA-4, drug name ipilimumab). Both of these treatments were administered intravenously (IV), with blood and CSF drawn prior to each dose when clinically indicated (see Methods) and sent for genomic analysis. Notably, both trials achieved primary endpoint (60% and 44% of patients were alive at 3 months after enrollment in the pembrolizumab and ipilimumab/nivolumab trials, respectively) and showed improved overall median survival (3.6 and 2.9 months in the pembrolizumab and ipilimumab/nivolumab trials, respectively) compared to historical controls^12,13^. Despite the promise shown in NCT02886585 and NCT02939300, questions remain regarding the utility and long-term efficacy of ICI to treat LMD. For example, it is not known whether the clinical benefit observed in these patients is strictly a result of the systemic effects of ICI administration, or whether these extend to the CNS. Additionally, the cellular and molecular features that underlie patient response have yet to be elucidated.

Here, we applied single-cell RNA-sequencing (scRNA-Seq) and cell-free DNA-sequencing (cfDNA-Seq) in conjunction with conventional clinical assays to serial CSF and peripheral blood leukocyte (PBL) samples from patients on these two ICI trials to: (1) describe the cellular composition of the LMD tumor microenvironment (TME); (2) assess inflammatory immune responses within the CSF; and, (3) identify potential factors informing the clinical courses observed in individual patients.

## Results

### scRNA-Seq of the LMD TME

**Fig. 1:**
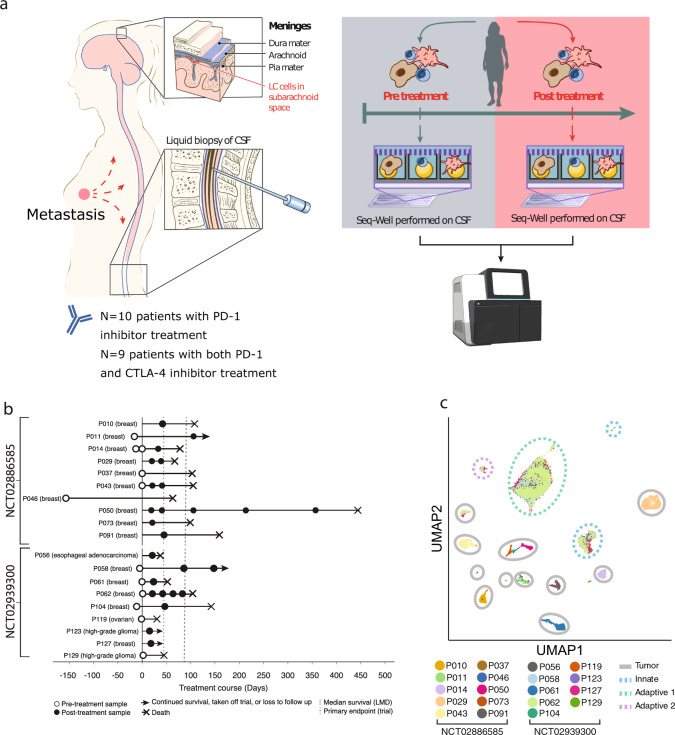
Development of a pipeline for scRNA/cfDNA from longitudinally sampled CSF samples before and after ICI therapy. **a** Schematic representation of the longitudinal sampling performed on patients in this study. **b** Longitudinal sampling overview from patients in each study, including trial primary endpoint (dashed line), and date of patient mortality, when known. **c** UMAP of single-cell transcriptomes from all captured CSF cells in both trials, colored by patient, with cell type of origin indicated.

We performed longitudinal high-throughput scRNA-Seq (Fig. 1a) on 12 pre-treatment and 25 post-treatment low-input CSF samples from 19 total patients enrolled in NCT02886585 and NCT02939300, including 9 patients sampled at multiple time points. After filtering for low quality cells, we retained 34,742 single cells from available clinical trial samples (Supplementary Fig. 1), which we further classified and visualized using dimensionality reduction by principal component analysis (PCA)^14^ and uniform manifold approximation and projection (UMAP; Methods)^14,15^. Our analyses reveal 17 distinct clusters, which we identified through differential gene expression (Supplementary Data 2) as adaptive immune cells (including T cells, immunoglobulin-expressing B cells), innate immune cells (including dendritic cells, monocytes, and macrophages), and non-immune cells (Fig. 1b, c). The non-immune cell clusters (*n* = 11) exhibited strong patient-specific representation while the immune clusters (*n* = 5) grouped by phenotype rather than patient (Supplementary Fig. 2), consistent with previous observations derived through scRNA-Seq of human tumors^16,17^.

After initially identifying non-immune clusters via unsupervised clustering, we confirmed their malignancy status for samples from NCT02886585 by inferring copy number variation (CNV) profiles for each cell and matching to DNA-based profiling results^17–19^. DNA-derived CNV profiles were obtained via whole exome sequencing (WES) of cell-free DNA (cfDNA) extracted from the CSF. Using these data, we confirmed that patient-specific non-immune clusters shared the inferred CNV profiles (as previously described) of their time-point matched cfDNA counterparts (Methods, Supplementary Fig. 3b). The proportions of tumor cells captured by Seq-Well for available time points (see Supplementary Data 1 for all available cytology reports) correlated significantly (Kendall’s т correlation = 0.62, *p* = 0.0027; Pearson correlation coefficient = 0.89, *p* = 1.8 × 10^−5^) with the reported tumor cell fraction detected by CytoSpin from CSF (Supplementary Fig. 3c). We additionally obtained PBL-derived scRNA data on 810 cells from patients P010, P043, P046, and P073 of trial NCT02886585 (Supplementary Fig. 4).

### CD8 + T Cells in CSF following intravenous ICI administration

**Fig. 2:**
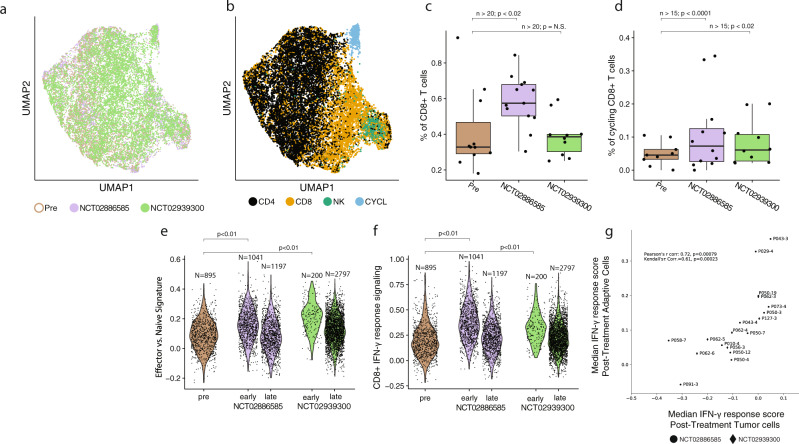
T cells in CSF exhibit strong differences in the expression of interferon-induced, cytotoxic, and exhaustion genes following ICI. **a**, **b** UMAP calculated over all T/NK cells (*n* = 16,954), colored by cohort (**a**) and canonical cell type (**b**) as identified via iterative subclustering (see Methods). **c** Percentage of CD8 + T Cells in pre-treatment, post-treatment cohort 1, and post-treatment cohort 2 samples; only samples with more than 20 T cells considered. **d** Proportion of CD8 + T cell cycling in pre-treatment, post-treatment cohort 1, and post-treatment cohort 2; only samples with more than 10 CD8 + T cells considered. **e** Effector vs naïve gene expression in pre-treatment, early post-treatment (<30 days post-treatment), and late post-treatment (≥30 days post-treatment) CD8 + T cells, *N* = 6,133 CD8 + T cells. **f** IFN-γ response in pre-treatment, early post-treatment (<30 days post-treatment), and late post-treatment (≥30 days post-treatment) CD8 + T cells. **g** Median IFN-γ response across samples in tumor cells vs CD8 + T Cells. In c–d data are represented as boxplots where the middle line is the median, the lower and upper hinges correspond to the first and third quartiles, the upper whisker extends from the hinge to the largest value no further than 1.5× IQR from the hinge (where IQR is the interquartile range) and the lower whisker extends from the hinge to the smallest value at most 1.5 × IQR of the hinge. In **c**–**f**, indicated p-values are two-sided, calculated from a Wilcoxon rank-sum test, and in figure g, *p*-value of the Kendall-Tau correlation is two-sided (see Methods).

We found that CD8 + T cells in the CSF are more abundant (NCT02886585) and proliferative (both trials) in samples treated with immune checkpoint inhibitors relative to untreated samples. We first performed unsupervised analysis of the T/NK cluster (Fig. 2a, b), and calculated the proportion of CD4 + T cells, CD8 + T cells, and NK cells (which can co-segregate with T cells during high-level analyses of scRNA-Seq data based on gene-expression similarity^20^) in each sample in our dataset (Supplementary Data 3-4). The proportion of CD8 + T cells in post-treatment CSF samples from NCT02886585 was significantly higher than in pre-treatment samples in evaluable samples (Cohen’s *d* = 0.87, Two-sided Wilcoxon’s rank-sum *p* = 0.03, *N* = 24 samples; 11 pre-treatment, 13 post-NCT02886585, see Methods), while there was no statistically significant difference in the proportion of CD8 + T cells in the post-treatment CSF samples of NCT02939300 relative to pre-treatment (Fig. 2c). Unsupervised analysis of the T cells also revealed a cluster of cells with increased expression of genes associated with proliferation, including *MKI67*, *BIRC5*, and *TOP2A*, among others^16,18,21^. Increased proliferation following ICI administration has previously been reported in the peripheral blood of patients undergoing systemic treatment^19,22,23,24^. We calculated the fraction of proliferating CD8 + T cells for each sample. Samples treated with ICIs had a significantly greater fraction of proliferating CD8 + T cells compared to untreated samples in evaluable samples (Cohen’s *d* = 0.62, 0.60; Two-sided Wilcoxon rank-sum *p* = 0.02, *N* = 21, 19, 9 pre-treatment, 12 post-NCT02886585, 10 post-NCT02939300; Fig. 2d, see Methods. Accompanying analysis of longitudinally matched samples across patients in Supplementary Fig. 5). These data suggest that the abundance and rates of proliferation of T Cells in the CSF increased post-treatment.

CD8 + T cells from samples treated with ICI transiently exhibited higher levels of genes associated with effector function and IFN-γ signaling relative to untreated samples, which were more naïve-like. Principal component analysis revealed that the first two principal components distinguished early post-treatment (<30 days since initial treatment) and pre-treatment samples (Supplementary Fig. 6). Loadings for these principal components were driven by genes associated with IFN-γ signaling as well as effector/naïve phenotypes of CD8 + T cells^25–27^. Indeed, we detected a significant increase in genes related to effector-like function and IFN-γ signaling (Fig. 2e, f; gene lists in Supplementary Data 5) in CD8 + T cells in recently treated (<30 days since initial treatment) samples vs. pre-treatment in both NCT02886585 (Cohen’s *d* = 0.91 and 0.65, *p* < 0.001 for both IFN-γ and effector/naïve signatures respectively) and NCT02939300 (Cohen’s *d* = 0.75 and 1.07, *p* < 0.001 for both IFN-γ and effector/naïve signatures, *N* = 6,133 CD8 + T cells). Furthermore, we found that the mean level of IFN-γ signaling in the T cells was strongly correlated with the mean IFN-γ response in tumor cells at the same time points (Kendall’s τ correlation = 0.67, *p* = 0.003, Fig. 2g), suggesting that inflammatory response is consistent across cell types in the same CSF sample.

### Longitudinal scRNA-seq reveals transient IFN-γ response and antigen presentation signatures following ICI administration

**Fig. 3:**
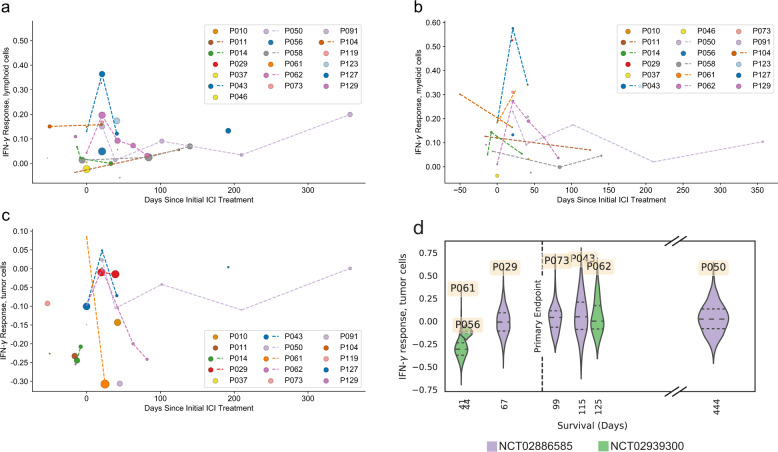
Acute immune response in CSF subsequent to intravenous ICI and relationship to survival. **a**–**c** Mean module score for IFN-γ response within samples over time points for lymphoid (**a**), myeloid (**b**), tumor cells (**c**). Samples from a single patient are connected with a dashed line. The size of markers is proportional to the number of relevant cells in a sample; only samples with more than 5 cells of the corresponding type are considered. Points at 0 days relative to ICI administration are pre-treatment. **d** Violin plots of IFN-γ response for tumor cells plotted against survival (time-on-trial), for samples taken <30 days after initial ICI administration. Medians and upper and lower quartiles are indicated in each violin plot by dashed lines.

IFN-γ response (Fig. 3a–c) and antigen presentation (Supplementary Fig. 7a–c) signatures exhibited transient upregulation immediately following ICI administration, which was observed across patients in multiple cell types. We found temporary elevation in the module scores for both signatures, with the maximum occurring at early time points (<30 days after initial administration; *p* = 0.00405, 0.00262, 0.0774 for IFN-γ response in lymphoid, innate, and tumor compartments respectively; *p* = 0.00406, 0.00871, 0.0938 for antigen presentation in lymphoid, innate, and tumor compartments respectively; Two-sided Wilcoxon rank-sum test throughout), and a significant reduction in the majority of these signatures at later time points (≥30 days after initial administration; *p* = 0.03212, 0.16491, 0.08648 for IFN-γ response in lymphoid, innate, and tumor compartments respectively; *p* = 0.022271, 0.049141, 0.032125 for antigen presentation in lymphoid, innate, and tumor compartments, respectively; Two-sided Wilcoxon rank-sum test throughout; *N* = 12 pre-treatment, *N* = 7 0–30 days post-treatment, *N* = 6 36+ days post-treatment for lymphoid, *N* = 12 pre-treatment, *N* = 8 0–30 days post-treatment, *N* = 7 36+ days post-treatment for innate, *N* = 11 pre-treatment and *N* = 7 0–30 days post-treatment, *N* = 6 36+ days post-treatment for tumor, see Methods). Taken together, these results suggest the onset of either an acute inflammatory response within the CSF to intravenously administered ICI or the infiltration of ICI-activated immune cells to this compartment, which may potentially explain the clinical efficacy of intravenous ICI against LMD in NCT02886585 and NCT02939300^12,13,28^.

### IFN-γ response and antigen presentation within malignant cells shortly (<30 days) after ICI administration correlates with time on trial

To see whether the previously described ICI response had prognostic value in this patient population, we compared IFN-γ response and antigen presentation within cell types with time-on trial. In total, 6 samples were obtained from individuals having received their initial ICI dose <30 days prior, for which we had known dates of death. We observed that in malignant cells there was a relationship between survival beyond primary endpoint and the mean module score of IFN-γ response (*p* = 0.0526, single-sided Wilcoxon rank-sum test^29^ Fig. 3d). This relation did not hold for non-malignant cells (Supplementary Fig. 8).

### Inflammatory signatures appear greater in the CSF than in the blood

We observed more pronounced inflammatory signatures (antigen presentation and IFN-γ response) in CSF than peripheral blood lymphoid and myeloid cells in post-treatment timepoint matched samples (*p*-values in Supplementary Data 6) while using a down-sampling procedure (Methods) to adjust for differences in cell quality. Furthermore, whereas we observed a significant increase in antigen presentation and IFN-γ response in CSF-derived innate and lymphoid immune cells in P043 immediately after treatment, this was not observed in PBL-derived innate and lymphoid immune cells (*p*-values provided in Supplementary Data 6). Moreover, while we observed a significant increase in M1-like phenotype^30,31^ in CSF-derived myeloid cells over time in P043 (*p*-values in Supplementary Data 6), we observed a decrease in the same signature in PBL-derived myeloid cells in that patient (*p*-values in Supplementary Data 6). This suggests that post-ICI inflammation in patients with LMD may be particularly elevated in the CNS, and that different compartments within the body may express divergent levels or stages of overall immune response, warranting further studies across multiple sites and timepoints to fully characterize patients’ overall response to therapy. Differential expression rankings (Methods) between PBL and CSF-derived lymphoid and myeloid cells are provided in Supplementary Data 7.

### An adaptive selection of a less immunogenic subclone coincides with transient response in one patient

**Fig. 4:**
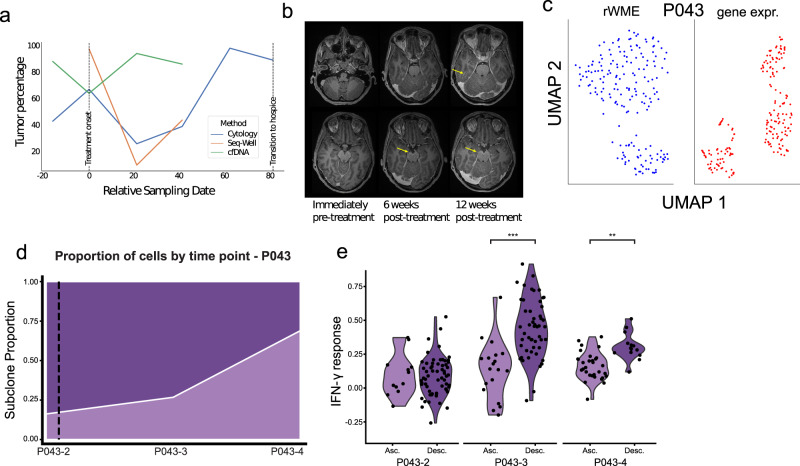
Longitudinal scRNA and cfDNA from P043 suggest the adaptive selection of a less-immunogenic over a more-immunogenic subclone. **a** Tumor fraction within CSF, as measured by Seq-Well and cytology, overlaid with tumor purity inferred by ABSOLUTE run on CSF-derived cfDNA. **b** MRI imagery at 0, 6, and 12 weeks relative to treatment; LMD-indicative enhancement indicated by red arrow. **c** Unsupervised clustering of inferred copy number profiles (left, see Methods) and expression (right) reveals intercellular heterogeneity, possibly explainable by the presence of subclones. **d** Relative proportions of subclones as a function of time. Darker purple and lighter purple denote the descendant and ascendant subclone, respectively. **e** IFN-γ response expression in subclones over time (***p = 0.001, **p = 0.01, Wilcoxon ranked-sum test, Cohen’s *d* = 1.4, *N* = 52 for descendent, 19 for ascendant at P043-3; Cohen’s *d* = 1.4, *N* = 14 for descendent, 31 for ascendant at P043-4). Darker purple and lighter purple denote the descendant and ascendant subclone, respectively.

We investigated cellular behavior underlying treatment response in a particular patient, P043, who showed unique clinical, phenotypic, and inferred CNV-based dynamics over the course of treatment. Three weeks following initial pembrolizumab administration, P043 showed a reduction in tumor burden according to both cytology and Seq-Well (Fig. 4a). At 6 weeks following initial ICI administration, and consistently from that point onward, the reported tumor cell fraction by cytology increased progressively until the patient came off study, with both Seq-Well and cytology showing an increase in tumor burden (Fig. 4a). This was accompanied by an interval increase in LMD-associated enhancement on the patient’s brain MRI at 6 weeks following treatment. At 12 weeks, malignant cytological fraction was above pre-treatment levels according to cytology, and MRI scans showed further LMD progression at 12 weeks, again indicative of LMD progression (Fig. 4a, b).

Unsupervised analysis of tumor cells from P043 revealed heterogeneity in both the gene expression and inferred copy number (rWME, see Methods) profiles that was suggestive of adaptive selection leading to acquired ICI resistance (see Fig. 4c). For all patients, we assessed the possibility of subclonal tumor heterogeneity^32–36^ by inferring single-cell CNV profiles^16,17,37^ via clustering in windowed mean expression (WME, see Methods) space. Visualizing these clusters with reduced dimensions, we found little structured CNV heterogeneity within 14 of the 15 evaluable patients (Supplementary Fig. 8). For example, in P029, we observed clusters in gene expression space (primarily attributable to cycling status) but not in inferred CNV space; in other patients (P014, P050, P061), the inferred CNV variation was limited, restricted to only a few loci, or sampled at only one time point. In P043, however, we detected the presence of two distinct copy number profile clusters, which suggested the presence of tumor subclones (Fig. 4c, Methods). Moreover, the fractional abundance of these two subclones shifted over time, with one clone lowly abundant pre-treatment and monotonically increasing at the expense of the other (Fig. 4d). We therefore hypothesized that a minority subclone was adaptively selected in P043 as a result of ICI administration.

We noted that regions of high divergence in inferred CNV between clusters corresponded to focal genomic amplifications distinguishing pre- and post-treatment cfDNA-derived copy-number profiles (Supplementary Fig. 9). Notably, the CNV-associated clusters were highly concordant with gene expression-derived clusters, suggesting correspondence between genetic and phenotypic heterogeneity in the tumor cells of P043 (Fig. 4c).

We plotted IFN-γ response scores for each cluster at the three time points measured for P043 (Fig. 4e). These data show that the descendent clone exhibited higher IFN-γ response genes at P043-3 (*p* < 2 × 10^−3^ for post-treatment time points, Two-sided Wilcoxon rank-sum test)–the time point immediately following treatment (Fig. 4e). In contrast, the ascendant clone exhibited consistently lower IFN-γ response across this trajectory, eventually predominating at the last time point (P043-4). Differences in antigen presentation between subclones (*p* = 0.01 at P043-3, *p* = 0.0015 at P043-4; *N* = 52 for descendent, 19 for ascendant at P043-3; *N* = 14 for descendent, 31 for ascendant at P043-4) are given in Supplementary Fig. 11d.

To support the subclonal hypothesis without relying on either inferred CNV profiles or unsupervised clustering thereof, we performed a supervised comparison of single-cell expression profiles at each time point to both the early and late cfDNA-derived WES copy ratios (Supplementary Fig. 11c). This analysis revealed that cells collected at P043-4 had gene expression profiles more concordant with the copy number profile calculated from cfDNA obtained at P043-4, whereas cells collected at P043-2 had gene expression profiles more concordant with the copy number profile from cfDNA obtained at P043-2. At post-treatment time points, cells with gene expression profiles correlating more strongly with copy number profiles from the later cfDNA (from P043-4) tended to have lower expression of IFN-γ response related genes while cells with gene expression profiles correlating more strongly with copy number profiles from the earlier cfDNA (from P043-2) tended to have higher expression of IFN-γ response related genes (Theil-Sen slope = −15.9 IFN-γ response/correlation difference for P043-3 and −8.79 IFN-γ response/correlation difference for P043-4).

Genes with large cfDNA-derived CNV difference between the time points P043-2 (enriched for the descendant subclone) and P043-4 (enriched for the ascendant subclone) represent hypothetical drivers for observed difference in immunogenicity between subclones. Supplementary Data 8 contains the top genes with the largest (respectively smallest) fold change in cfDNA-derived copy ratio at P043-2 and P043-4, and the corresponding mean expressions of those genes in the ascendant and descendant subclones; *RAD21*, for example, which has been reported to predict poor prognosis^38,39^, is copied roughly 5 times more in the cfDNA at P043-4, and is roughly 3 times more highly expressed in the ascendant subclone when controlling for time point (i.e., at time point P043-3).

## Discussion

Here, we have used low-input profiling techniques to perform scRNA-Seq characterization of cell types in the liquid component of the human LMD TME. We catalog the presence of tumor cells, lymphoid cells, and myeloid cells, examining shifts in their abundance and phenotype in conjunction with two clinical trials of ICI efficacy. These highly-resolved data enable further study of LMD- and treatment-associated phenomena that are unique to the CSF both within and across patients.

We calculated statistically significant increases in the abundance and outgrowth of CD8 + T cells in the CSF following ICI administration relative to pre-treatment in NCT02886585. Additionally, we detected higher overall levels of IFN-γ signaling and cytotoxicity in CD8 + T cells post-treatment in both trials. These results suggest that intravenous ICI administration modulates the immune microenvironment in the CSF of a subset of patients in these clinical trials, and that this may have been associated with observed clinical benefit.

We detect suggestive evidence that the magnitude of the initial inflammatory response among malignant cells may have prognostic value, warranting further investigation. Investigation of IFN-γ response and antigen processing signatures in the tumor compartments of patients in these trials reveals a distinct increase across patients immediately following the first ICI dose followed by a steady decrease over time. While this observation is consistent with reports of anti-PD-1 administration in the peripheral blood^40,41^, it also illustrates a potential limitation in ICI efficacy for LMD. Additionally, these results underscore the importance of sampling time, as well as the value of longitudinal profiling, in scRNA-seq studies of response to therapies, as these responses are liable to exhibit transient transcriptional effects; more time-resolved sampling may be necessary to properly characterize the dynamic phenotypic processes presented here.

Finally, in a particular patient, we note evidence suggesting the existence of subclones that might underlie the transient response to ICI observed in that individual. We use that evidence to propose hypothetical drivers of increased or decreased immunogenicity^42^.

These results collectively support the findings of Brastianos et al.^12^ and Brastianos et al.,^13^—namely, that ICI shows clinical efficacy in patients with LMD. Additionally, we observe strong compartmental and temporal variation in inflammatory signatures in the post-therapy TME, with significant implications for future study design in LMD and in other cancer types.

Due to the limited input volumes obtainable from clinical CSF samples, we were not able to obtain longitudinal data from all patients at all time points. Moreover, the diversity of the cohort—containing diversity in both primary histology and histological subtype—recommends that follow-up studies control for these factors, so as to confirm whether the biomarkers of response suggested above have clinical utility. Future work with larger cohort sizes, and more frequent longitudinal—as well as multi-site—sampling will enable the characterization of genotypic factors in ICI response in LMD, support comparisons of the effects of multiple vs. single-drug treatments, and further test and refine the prognostic biomarkers suggested in this work.

## Methods

### Study design: patients

These clinical trials (Clinicaltrials.gov identifier NCT02886585 and NCT02939300) were designed by the principal investigators and the Dana-Farber Harvard Cancer Center (DF/HCC) Institutional review board approved the protocol. The study was designed by the principal investigators and conducted in accordance with the provision of the Declaration of Helsinki and Good Clinical Practice guidelines. The Dana-Farber/Harvard Cancer Center institutional review board approved the protocol. All patients provided informed consent. Eligible patients had histologically confirmed disease from any solid tumor and LMD defined by positive CSF cytology for malignant cells. Other key inclusion criteria included the following: an ECOG performance status ≤2, normal organ and marrow function, and a stable dose of dexamethasone of 2 mg or less for 7 days prior to initiation of treatment. Given the frequent occurrence of neurologic symptoms (e.g. headaches) associated with LMD, 11/20 patients included in this study were on a steroid regimen at the time of enrollment and 10/20 patients were treated with steroids while receiving ICI^13^. Written informed consent was obtained for all participants.

Further details of the subjects’ clinical courses, including cytology, steroid dosage, and Ommaya shunt status, are provided in Supplementary Data 1.

### Study design: treatment and endpoints

These studies were designed as open-label, single arm Phase-II clinical trials to evaluate ICI in patients with LMD of any histology. For the first trial (NCT02886585), patients with CNS metastases were enrolled across multiple cohorts. Cohorts A, B, and D include patients with parenchymal brain metastases. The LMD cohort was Cohort C of this study. Pembrolizumab was administered intravenously at 200 mg every 3 weeks until disease progression, death, or grade 3–4 toxicity. A brain MRI and CT chest/abdomen/pelvis were obtained every 6 weeks for restaging purposes. The primary endpoint of the LMD cohort was the rate of overall survival at 3 months (OS3). 11 patients with LMD enrolled to NCT02886585 were included for single-cell analysis; 4 of whom had serum and CSF sampling at multiple time points during treatment. All 11 patients had metastatic breast cancer (Supplementary Data 1).

Treatment in the second trial (NCT02939300) consisted of ipilimumab and nivolumab. Ipilimumab and nivolumab were administered intravenously every 3 weeks for 4 doses. Afterwards, ipilimumab was given every 6 weeks and nivolumab was given every 2 weeks (for non-small cell lung cancer and head and neck cancer) or 4 weeks (for all other malignancies). Treatment was continued until disease progression, death, or grade 3–4 toxicity. A brain MRI and CT chest/abdomen/pelvis were obtained every 6 weeks for restaging purposes. The primary endpoint was OS3. 9 patients on trial were included for single-cell analysis; 5 of whom had serum and CSF sampling at multiple time points during treatment. 5 patients had metastatic breast cancer, 2 patients had high-grade glioma, 1 patient had ovarian cancer, and 1 patient had esophageal cancer.

Further details of the subjects’ clinical courses, including cytology, steroid status, and Ommaya/VP shunt status are provided in Supplementary Data 1.

### CSF cell extraction

Cerebrospinal fluid (CSF) from patients was extracted via an Ommaya reservoir or ventriculoperitoneal shunt (VPS) as part of clinical care. CSF not required for clinical testing was spun at 800 G for 5 min to pellet cells, and resuspended in PBS (ThermoFisher 10010023, Ca/Mg-free). Red blood cells (RBCs) were lysed using ACK lysis buffer (ThermoFisher A1049201) for 4 min on ice to remove RBCs. Cells were then washed with sterile PBS and spun down at 800 G for 5 min, resuspended as a single-cell suspension in RPMI (Corning) with 10% FBS (ThermoFisher 10082-147) for subsequent scRNA-Seq. Cytology was performed whenever possible from available CSF; cytology results for all available samples are given in Supplementary Data 1.

### Peripheral blood lymphocyte (PBL) extraction

A 10 cc tube of blood was collected and processed within 3 hours of blood draw. 15 mL of Lymphoprep (STEMCELL Technologies, Catalog #07801) and 10 mL of phosphate-buffered saline was added to the blood. This mixture was then centrifuged at 1200 G for 12 minutes. The supernatant was then poured out and 10 mL of phosphate-buffered saline was added. This mixture was centrifuged a second time at 500 G for 5 min. This supernatant was poured out and 1 mL of CryoStor CS10, Cryopreservation Freeze Media (STEMCELL Technologies, Catalog MSPP-07930) was added to the pellet. This mixture was frozen at −80 °C, then later thawed on ice, then to room temperature, then processed using Seq-Well as described below for CSF-derived cells.

### Extraction and sequencing of cell-free DNA

For blood, a 10 cc tube was first centrifuged at 500 g for 10 min. Afterwards, the supernatant was extracted and then centrifuged again at 1000 g for 10 min. The second supernatant was then used for serum cell-free DNA extraction and sequencing. For CSF samples, a 3 cc tube of sample was centrifuged at 400 G for 5 min.

Extraction of cell-free DNA from banked plasma and centrifuged CSF was done using an automated liquid handler at the Broad Institute’s Blood Biopsy Lab. Sequencing was then conducted by the Broad Institute’s core facility.

### scRNA-Seq with Seq-Well

Resuspended CSF cells were profiled using the Seq-Well platform for massively parallel, high-throughput scRNA-seq for low-input clinical samples. A complete description of methods is available online^43^. A complete list of primers described in Gierahn et al.^43^ is additionally provided in Supplementary Data 10. Briefly, cells from each CSF sample were manually counted (Bal Supply 808CI) and loaded onto one array preloaded with barcoded mRNA capture beads (ChemGenes). All samples retained fewer than 10,000 cells with the exception of two (CSF029-4 & DFCI010-4; ~100,000 cells). Thus, all available cells were loaded onto a single array, except CSF029-4 and DFCI010-4 where ~10,000 cells were loaded. The loaded arrays containing cells and uniquely barcoded oligo-dT beads were then sealed using a polycarbonate membrane with a pore size of 0.01 μm, which allows for the exchange of buffers but retains biological molecules confined within each nanowell. Subsequent buffer exchanges facilitated cell lysis, transcript hybridization, and bead recovery before performing reverse transcription *en masse*. Following reverse transcription using Maxima H Minus Reverse Transcriptase (ThermoFisher EP0753) and an Exonuclease I treatment (NewEngland Biolabs M0293L) to remove excess primers, PCR amplification was carried out using KAPA HiFi PCR Mastermix (Kapa Biosystems KK2602) with approximately 2,000 beads per 50 μl reaction volume. Libraries were then pooled into one tube (with the exception of CSF014-4, CSF029-4, CSF046-2, CSF104-1, CSF104-3, and CSF123-4, which were pooled to two tubes) and purified using Agencourt AMPure XP beads (Beckman Coulter, A63881) by a 0.6X SPRI followed by a 0.8X SPRI and quantified using Qubit hsDNA Assay (Thermo Fisher Q32854). The quality of each WTA product was assessed using the Agilent hsD5000 Screen Tape System (Agilent Genomics) with an expected peak ranging between 800 and 1500 bp tailing off to beyond 5000 bp, and a small or non-existent primer peak (~100–200 bp).

3′ digital gene expression (DGE) libraries were constructed using the Nextera XT DNA tagmentation method (Illumina FC-131-1096) using index primers as described in Gierahn et al.^28^. Loaded samples ranged from 600 to 2,000 pg of pooled cDNA, depending on the peak distribution of the WTA product for the sample. Tagmented and amplified sequences were purified at a 0.6× SPRI ratio followed by a 0.9X SPRI yielding library sizes with an average distribution of 400–750 base pairs in length as determined using the Agilent hsD1000 Screen Tape System (Agilent Genomics). Samples DFCI010-4, CSF011-1, CSF011-7, CSF014-1, CSF014-2, and CSF014-4, CSF029-2, CSF029-5, DFCI037-1, CSF046-2, CSF050-4, CSF050-7, and CSF073-4 were sequenced using an Illumina 75 Cycle NextSeq500/550v2 kit (Illumina 20024906) at a final concentration of 2.2–2.8 pM. Samples CSF029-4, CSF043-2, CSF043-3, CSF043-4, CSF050-3, CSF050-12, CSF050-19, DFCI056-3, DFCI058-1, DFCI058-5, DFCI058-7, DFCI061-1, DFCI061-2, DFCI062-2, DFCI062-3, DFCI062-4, DFCI062-5, DFCI062-6, CSF091-3, CSF104-1, CSF104-3, CSF119-1, CSF123-4, CSF127-3, and CSF129-1 were sequenced using an Illumina 100 Cycle NovaSeq6000S kit (Illumina 20027464). The read structure in both cases was paired end with read 1 starting from a custom read 1 primer containing 20 bases with a 12-bp cell barcode and 8-bp unique molecular identifier (UMI) and read 2 containing 50 bases of transcript information.

### Alignment & Pre-processing of scRNA-Seq data

Read alignment was performed as in Macosko et al.^21^. In brief, for each Illumina sequencing run, raw sequencing data were converted to demultiplexed FASTQ files using bcl2fastq2 based on Nextera N700 & N500 indices corresponding to individual samples/arrays. Reads were then aligned to hg19 genome using the dropseq_tools v2.1.0 pipeline maintained by the Broad Institute using standard settings. Individual reads were tagged according to the 12-bp barcode sequenced and the 8-bp UMI contained in Read 1 of each fragment. Following alignment, reads were binned onto 12-bp cell barcodes and collapsed by their 8-bp UMI with a hamming distance correction of 1. DGE matrices (genes-by-barcode) for each sample were obtained from quality filtered and mapped reads, with an automatically determined threshold for barcode count.

DGEs from each sample were individually culled and inspected by unsupervised analysis before inclusion into the full analysis by a combination previously described methods^29,30^. Each barcode was initially scored on: (1) average expression of a list of curated housekeeping genes (Supplementary Data 5) and (2) complexity, estimated by the total number of genes detected. All sequenced samples were cut to exclude barcodes with low complexity/housekeeping gene expression (400 gene complexity cutoff, housekeeping gene expression cutoff of 1.6 log_2_(tp10k)). Each sample was then inspected using unsupervised analysis to further identify and curate potential analyzable single cells. Individual arrays were analyzed to determine the extent of cross-cell type gene expression contamination. Minimal cross-cell type gene expression contamination existed between immune subsets, and select barcodes were filtered out according to cross expression of marker genes from other immune subsets. Further restrictive analyses incorporating lowered complexity thresholds and count-based downsampling was performed to control for technical confounders wherever relevant. Following curation, all samples were combined and genes expressed in at least 1% of the remaining barcodes were retained (in the case of WME calculations used in Fig. 4c we retained genes expressed in at least 0.0875% of remaining barcodes; in the case of WME calculations used in Supplementary Fig. 9, we retained all genes). Consecutive samples from the same patient were combined by assigning zeros to all undetected genes per sample and concatenating columns. miRNA and T cell receptor chain genes were subset and saved before cutting genes to ensure information was not lost. This curated, UMI-collapsed data was then normalized to 10,000 UMIs per barcode (tp10k) and log_2_-normalized before being input into Seurat^14^ v2.3.4 (https://github.com/satijalab/seurat) for further analysis. This yielded a Seurat object of 34,742 single cells and 8,156 genes, with different genes being used for more specific analysis (such as T cell analysis). The 37 individually sampled time points averaged 890.8 cells per sample with a range between 103 cells and 1,946 cells (Supplementary Data 1).

### Alignment & Pre-processing of PBL-derived scRNA-Seq data

Read alignment was performed identically as with CSF-derived scRNA-Seq data. Barcodes were selected from DGEs with a 200 gene complexity cutoff. Unsupervised analysis was performed jointly with CSF-derived cells, with the same complexity cutoff, from patients with PBL-derived data (P010, P043, P046, P073). All genes were retained. In total, 810 PBL-derived immune cells were detected.

For PBL vs CSF comparison, to account for differences in cell qualities between these samples, all cells had their total UMI count adjusted to 500 (the approximate mean UMI count of PBL-derived cells with complexity greater than 200). This was done by randomly selecting 500 UMIs for each cell, with sampling probabilities given by the pre-adjusted UMI count for that gene, in that cell. This data was then normalized to 10,000 UMIs per barcode (tp10k) and log_2_-normalized. Differential expression and module score calculations were performed as with CSF-derived scRNA-Seq data.

### Unsupervised transcriptomic analysis

Before performing dimensionality reduction, a list of the 2,359 most variable and highly expressed genes was generated by including genes with an average normalized and scaled expression value greater than 0.1 and with a dispersion (variance/mean) greater than 0.1. We then performed principal component analysis (PCA) over the list of variable genes. For both uniform manifold approximation and projection (UMAP) and SNN (shared nearest neighbor) clustering, we used the first 30 principal components. We used FindClusters within Seurat (which utilizes a SNN modularity optimization-based clustering algorithm) with a resolution of 0.7 and UMAP with minimum distance of 0.2 and number of neighbors of 50 to identify 27 clusters across the 37 input samples. 11 of these clusters were collapsed due to gene expression similarity evaluated by prior biological knowledge (7 extraneous divisions in cluster 0, 1 extraneous division in cluster 1, 2 extraneous divisions in cluster 3, and 1 extraneous division in cluster 4) to arrive at 17 total biological clusters across all samples.

Dimensional reduction on data from the CD8 + T cells and myeloid cells alone was similarly performed using PCA followed by UMAP and SNN clustering, all implemented in Seurat. For CD8 + T cells, principal components 1-6 were used with UMAP parameters of minimum distance 0.3 and number of neighbors 20; a resolution of 0.4 was used to identify clusters.

### Cell type identification and within cell type analysis

To identify genes that defined each cluster, we performed differential expression using the “bimod” test implemented with the FindMarkers function in Seurat based on a likelihood ratio test designed for single-cell differential expression incorporating both a discrete and continuous component. Thresholds were set at an average log-fold difference 0.2 and adjusted *p*-value (Bonferroni) less than 0.05. Top marker genes with high specificity were used to classify cell clusters into cell types (Supplementary Data 2,4) based on literature precedent. Two closely related clusters (T/NK clusters) were merged based on biological curation and analysis of hierarchical cluster trees yielding the twelve unique clusters. For T cells, we subclustered first on treatment condition, as we found that the original clusters were dependent on this metadata. The process used for clustering and subset identification was adapted for each iteration of clustering to optimize the parameters of variable genes, principal components, and resolution of clusters desired. Following identification of canonical subsets – CD4 + T cells, CD8 + T cells, and NK cells – these identities were assigned to the main T/NK cluster cells.

One cluster, cluster 15, containing 50 cells was not classified as immune or malignant. All cells in this cluster came from sample CSF011-1 and upregulated genes associated with neuronal expression, and this cluster was classified as “other.”

NK cell clusters within the pre-treatment and post-treatment T/NK groups were annotated based on expression of *CD2* and *FCRG3A* (*CD16*), lack of expression of *CD3* genes (*CD3D*, *CD3E*, *CD3G*).

The plasmacytoid DC (pDC) and conventional DC (cDC) clusters were distinguished from the other innate cells as dendritic cells, and then the differentially expressed genes between the two clusters were enriched using GSEA. The top GSEA hits on both gene lists distinguished cDCs and pDCs (Supplementary Data 9).

Comparisons of abundance of T cells were made across time points with at least 20 T cells detected (34 of 37 time points). Comparisons of proliferation of CD8 + T cells were made across time points with at least 15 CD8 + T cells detected (31 of 37 time points).

### Differential expression and scoring over gene sets

To identify differentially expressed genes within cell types and subtypes across treated and untreated conditions, we again used the ‘bimod’ setting in FindMarkers implemented in Seurat. To determine the scores of gene sets and pathways, such as IFN-γ response and antigen processing, we used the ‘AddModuleScore’ function in Seurat to construct a mean score of supplied genes subtracting a background score constructed from a random selection of genes in bins of average expression across all cells. When comparing scores within a specific subset of cells, AddModuleScore was constructed only over that subset, and recalculated if the subset was further partitioned. Tumor cell scores were calculated both across all patients (to compare pre-treatment and post-treatment time points across patients) and within individual patients (to compare across time points within patients). For specific comparisons of AddModuleScore-derived signatures with large differences in complexity between groups of cells, an upper complexity threshold and count-based downsampling were used to examine the possibility of complexity-confounded effects. No such effects were observed in comparing between tumor cells across patient and within patient.

### IFN-γ Response, Antigen Presentation, and Exhaustion Signatures

IFN-γ response signature and exhaustion signatures were obtained from GSEA (HALLMARK_INTERFERON_GAMMA_RESPONSE, various signatures from Wherry et al. 2007), provided in Supplementary Data 5. Antigen presentation signature was compiled following a search of the literature and is provided in Supplementary Data 5.

### Inferred CNV analysis of Patient 043

To more accurately infer CNV patterns in high-complexity (complexity > 1000) tumor cells with sub-chromosomal resolution, we group genes into fixed length windows of 200 genes consecutive along the genome, removing from consideration those genes in the uppermost decile of dropout rate, as well as all immunoglobulin genes. All possible windows were considered where all included genes reside on the same chromosome. We converted the log (TP10k + 1) gene expression profiles to TP10k ones. We then took the mean TP10k expression over genes in a window, neglecting the highest 5 gene expressions in that window. This vector of values is hereafter referred to as the unnormalized Windowed Mean Expression (uWME).

Having identified the malignant cells for each patient, we additionally computed a normalized version of the uWME as follows: the uWME from all patients’ non-malignant cells were averaged for each window across patients. HLA-* and associated genes on the 6p arm exhibited particularly strong hematopoietic expression; therefore, the means of these windows were imputed with the mean (windows) of the mean (patients) WME for all other windows. These values we refer to as the mean non-malignant uWME.

We normalize uWME for malignant cells by dividing the window uWME by the mean non-malignant uWME for each window, hereafter referred to as Windowed Mean Expression (WME).

To reduce possible confounding factors due to experimental or batch effects during subsequent clustering analysis, we converted the WME values in each single cell to ranks, hereafter referred to as the ranked, normalized WME (rWME). PCA and UMAP were performed on the rWME using the first 50 principal components of all tumor cells. In this P043, the UMAP-obtained clustering was concordant with that achieved via agglomerative clustering. To perform this clustering, we used as a distance metric 1-τ_K_, where τ_K_ is the Kendall’s т coefficient between the WME of all pairs of cells. Agglomerative clustering was performed with a weighted linkage to obtain four clusters; two clusters contained single cells, and two other clusters contained 128 and 62 cells and were denoted descendant and ascendant respectively.

To support the subclonal hypothesis without relying on either inferred CNV profiles or unsupervised clustering thereof, we performed a supervised comparison of single-cell expression profiles at each time point to both the early and late cfDNA-derived WES copy ratios (Fig. 4d). We calculate the Kendall’s т correlation for all genes’ total copy ratio and single-cell expression, for all single cells. Then we calculate the difference in correlation for all single cells when using total copy ratio from time point 4 (late) vs. time point 2 (early). We observe that CSF043-2 single cells exhibit correlations more similar to WES from time point 2, and that CSF043-4 single cells exhibit correlations more similar to WES from time point 4. At CSF043-3, we observe bimodality in the distribution of the difference of Kendall’s т correlations. Additionally, we observe that single cells derived from post-treatment time points (CSF043-3 and CSF043-4) exhibit anti-correlation between their IFN-γ response score (Fig. 4d) and the difference in Kendall’s т correlations between total copy ratios derived from WES at time point 4 vs. time point 2.

We note that the relative populations of the two identified clusters in P043 varied significantly across time (Fig. 4e). We plotted, for each gene, the mean purity corrected tCR vs change in the WME between all possible pairs of time points. The purity corrected tCR has the following form:

1 $${{{{{{\rm{tCR}}}}}}}_{{{{{{\rm{corrected}}}}}}}=\frac{{{{{{{\rm{tCR}}}}}}}_{{{{{{\rm{observed}}}}}}}-\left(1-p\right)\times {{{{{{\rm{tCR}}}}}}}_{{{{{{\rm{germline}}}}}}}}{p}=\frac{{{{{{{\rm{tCR}}}}}}}_{{{{{{\rm{observed}}}}}}}-\left(1-p\right)}{p}$$

where *p* is purity of sample calculated by ABSOLUTE^44^ and tCR_germline_ = 1.

This relationship is demonstrated in Supplementary Fig. 11, showing that the windowed expressional change between these clusters is concordant with the change in WES-derived tCR between any two time points. This concordance is robust to considering only the cells obtained at time point 3 (i.e., the correlated changes in single-cell expression and cfDNA-derived CNV profile cannot be attributed to batch effects confounding the observed scRNA-seq profiles).

Windowed-mean expression results were compared to the InferCNV R package from the Broad Institute, and broad amplifications and deletions were concordant between the two approaches (Supplementary Fig. 11a,b).

### Statistical analyses

Statistical analyses of differential expression were performed using Seurat v2.3.4 implemented in RStudio. All statistical tests of distributions, cluster diversity, and change in representation, etc. were performed using base R packages implemented in RStudio. All statistical tests of gene set enrichment were performed using piano v1.22.0 and implemented in RStudio for all except enrichments of cluster markers for the full dataset, which was implemented in R. All violin plots and boxplots were generated using ggplot2 without modifications to smoothing or density. Boxplot rectangles encompass the 25^th^ to 75^th^ percentile with outliers as individual points above and below the rectangle. Overlapping genes between IFN response and antigen processing signatures were removed from both before utilization.

As scores followed non-normal distributions as tested for using a Lilliefors normality test, we used a Wilcoxon rank-sum test where indicated for determining statistical significance. For scores in single-cell data, we report effect sizes in addition to statistical significance as an additional metric to capture the magnitude of the effect observed. The calculation was performed as Cohen’s *d* where: effect size *d* = (Mean_1_-Mean_2_)/(s.d. pooled). In Supplementary Fig. 5, the calculation of Cohen’s *d* was modified to *d*_pair _= (Mean_1_-Mean_2_)/(s.d._2_), where the difference in means is normalized by the standard deviation of the pre-treatment group. All *p*-values subject to the multiple comparisons problem (such as marker identification by differential expression) were adjusted by Bonferroni correction. Wilcoxon rank-sum tests were calculated via the R command wilcox.test. Related Student’s t-test p-values were computed in python 3.7.7 using the function scipy.stats.ttest_rel from scipy v1.5.4. Theil-Sen slopes were computed in python 3.7.7 using the function scipy.stats.theilslopes from scipy v1.5.4. Kendall-tau correlations and associated p-values were computed using the function scipy.stats.kendalltau from scipy v1.5.4.

### Reporting summary

Further information on research design is available in the Nature Research Reporting Summary linked to this article.

## Supplementary information

Supplementary Information

Descriptions of Additional Supplementary Files

Supplementary Data 1

Supplementary Data 2

Supplementary Data 3

Supplementary Data 4

Supplementary Data 5

Supplementary Data 6

Supplementary Data 7

Supplementary Data 8

Supplementary Data 9

Supplementary Data 10

Reporting Summary

## Data Availability

The genes-by-cells matrix and associated metadata generated from CSF draws and analyzed during the current study is available via the single-cell portal: https://singlecell.broadinstitute.org/single_cell/study/SCP1332/genomic-and-transcriptomic-correlates-of-immunotherapy-response-within-the-tumor-microenvironment-of-leptomeningeal-metastases. Raw data have been deposited in the dbGaP database under accession code phs002416.v1.p1 [https://www.ncbi.nlm.nih.gov/projects/gap/cgi-bin/study.cgi?study_id=phs002416.v1.p1] and the data are available under restricted access. Raw data are also available on the Broad Data Use and Oversight System (DUOS) through the accession codes DUOS-000131 and DUOS-000132: https://duos.broadinstitute.org/. The remaining data are available within the Article, Supplementary Information or Source Data file. Source data are provided with this paper.

## Source data

Source Data

## Code availability

Panopticon v0.1 has been made publicly available (https://github.com/scyrusm/panopticon/)^45^. R or python notebooks details of figure creation available upon reasonable request.
