# Author Correction: Genomic and transcriptomic correlates of immunotherapy response within the tumor microenvironment of leptomeningeal metastases

**DOI:** 10.1038/s41467-021-27207-6

**Published:** 2021-11-25

**Authors:** Sanjay M. Prakadan, Christopher A. Alvarez-Breckenridge, Samuel C. Markson, Albert E. Kim, Robert H. Klein, Naema Nayyar, Andrew W. Navia, Benjamin M. Kuter, Kellie E. Kolb, Ivanna Bihun, Joana L. Mora, Mia Solana Bertalan, Brian Shaw, Michael White, Alexander Kaplan, Jackson H. Stocking, Marc H. Wadsworth, Eudocia Q. Lee, Ugonma Chukwueke, Nancy Wang, Megha Subramanian, Denisse Rotem, Daniel P. Cahill, Viktor A. Adalsteinsson, Jeffrey W. Miller, Ryan J. Sullivan, Scott L. Carter, Priscilla K. Brastianos, Alex K. Shalek

**Affiliations:** 1 Department of Chemistry, Massachusetts Institute of Technology, Cambridge, MA USA; 2 Institute for Medical Engineering & Science, Massachusetts Institute of Technology, Cambridge, MA USA; 3 Broad Institute, Harvard University & Massachusetts Institute of Technology, Cambridge, MA USA; 4 Koch Institute for Integrative Cancer Research, Massachusetts Institute of Technology, Cambridge, MA USA; 5 Ragon Institute, Harvard University, Massachusetts Institute of Technology, & Massachusetts General Hospital, Cambridge, MA USA; 6 Department of Neurosurgery, Harvard Medical School & Massachusetts General Hospital, Boston, MA USA; 7 Division of Computational Biology, Dana-Farber Cancer Institute, Boston, MA USA; 8 Department of Medicine, Harvard Medical School & Massachusetts General Hospital, Boston, MA USA; 9 Massachusetts General Hospital Cancer Center, Boston, MA USA; 10 Division of Neuro-Oncology, Dana-Farber Cancer Institute, Boston, MA USA; 11 Department of Biostatistics, Harvard TH Chan School of Public Health, Boston, MA USA; 12 Division of Health Science & Technology, Harvard Medical School, Cambridge, MA USA; 13 Program in Computational & Systems Biology, Massachusetts Institute of Technology, Cambridge, MA USA

**Keywords:** Genomics, Cancer immunotherapy, CNS cancer, Metastasis

Correction to: *Nature Communications* 10.1038/s41467-021-25860-5, published online 12 October 2021.

This article contained errors in Fig. 1b and Supplementary Data 1. In Fig. 1b the sample P061_2 was incorrectly presented with an icon denoting "pre-treatment" status. In the “Results” section, the related sentence "We performed longitudinal high-throughput scRNA-Seq (Figure 1a) on 13 pre-treatment and 24 post-treatment low-input CSF samples" was also incorrect. The sentence should have read: “We performed longitudinal high-throughput scRNA-Seq (Figure 1a) on 12 pre-treatment and 25 post-treatment low-input CSF samples”. The correct samples and treatment timing were used elsewhere in the manuscript. These errors have been corrected in the PDF and HTML versions of the Article.

The original version of Supplementary Data 1 showed "days since treatment" for sample P129_1 indicated as "350"; however, the sample was obtained 15 days prior to therapy. This error has been corrected in Updated Supplementary Data 1.

## Supplementary information

Updated Supplementary Data 1

