# Supplementary material for: Genomic and transcriptomic correlates of immunotherapy response within the tumor microenvironment of leptomeningeal metastases
Source: Nat Commun. 2021 Oct 12;12:5955. doi: 10.1038/s41467-021-25860-5 (PMC8511044; doi:10.1038/s41467-021-25860-5)
Supplement: Supplementary file 1 — Supplementary Information [file 41467_2021_25860_MOESM1_ESM.docx]

**Supplementary Figures and Tables**


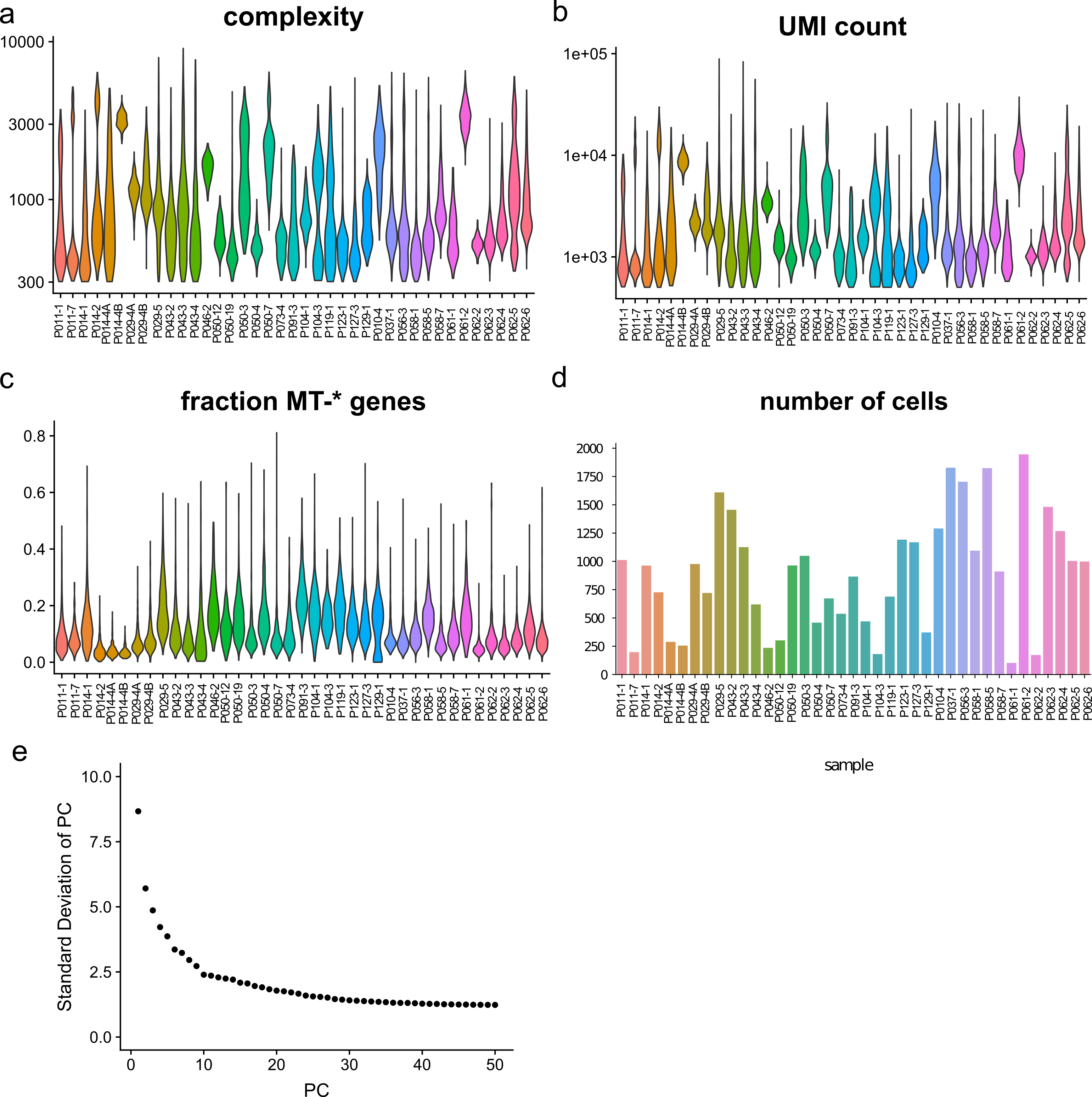


**Supplementary Fig. 1. Quality metrics of scRNA data.** Violin plots of a) complexity (log-scale), b) UMI count (log-scale), c) fraction of UMI counts aligning to the mitochondrial genome. d) bar plot of number of cells across samples. e) standard deviation of principal components of log_2_(TP10k+1) transformed UMI counts.


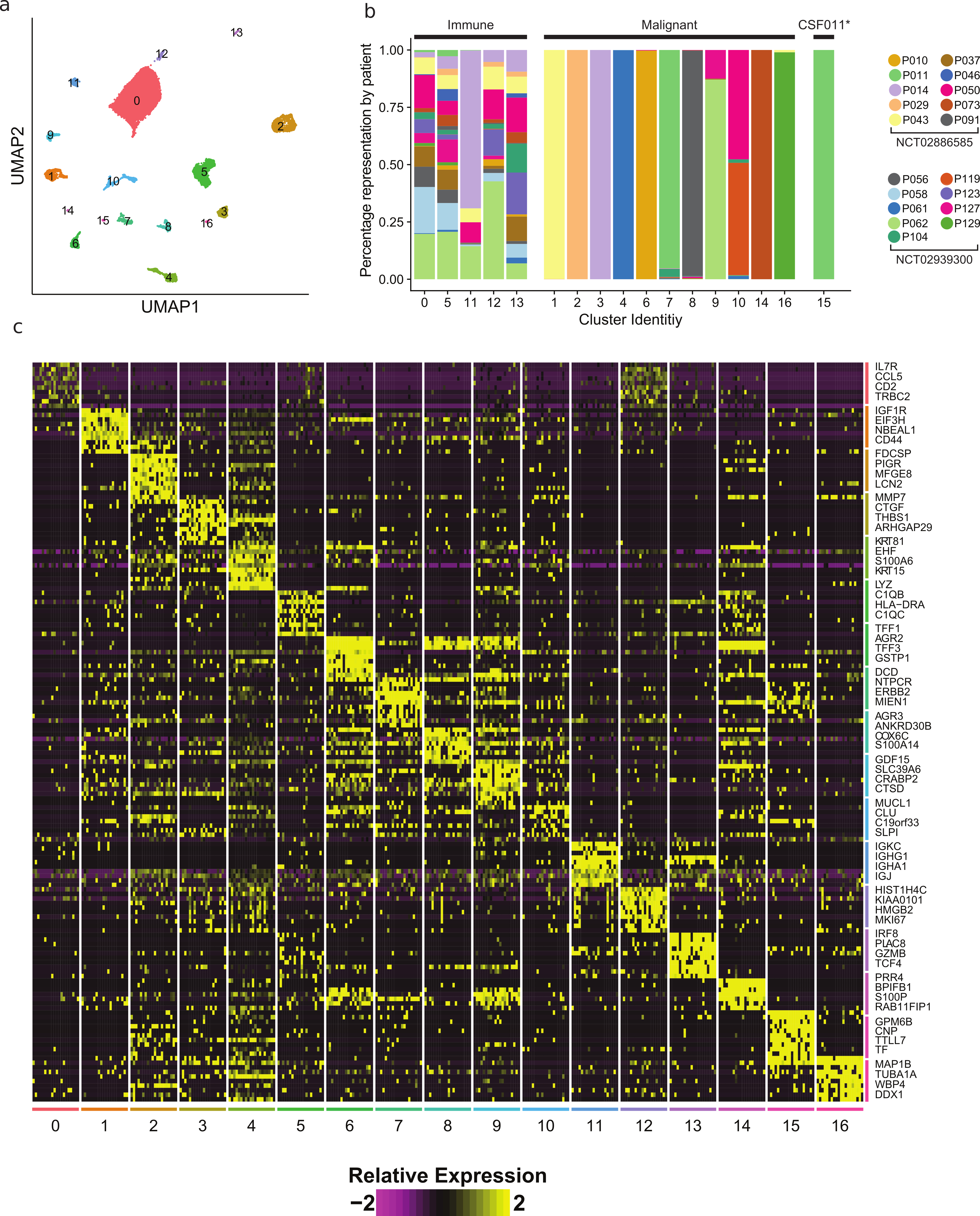


**Supplementary Fig. 2: Cluster identification across all single cell transcriptomes**. a) UMAP of 34,742 single cells colored by SNN-derived clusters across the top 30 principal components. b) Single-cell clusters comprised of patient-of-origin and delineated by cell type (immune, malignant, and other, see Methods). c) Heatmap of marker genes for each of 17 clusters identified in (a). For visualization, 10 single cells from each cluster were chosen as representative, and the top 4 marker genes are listed in the heatmap.


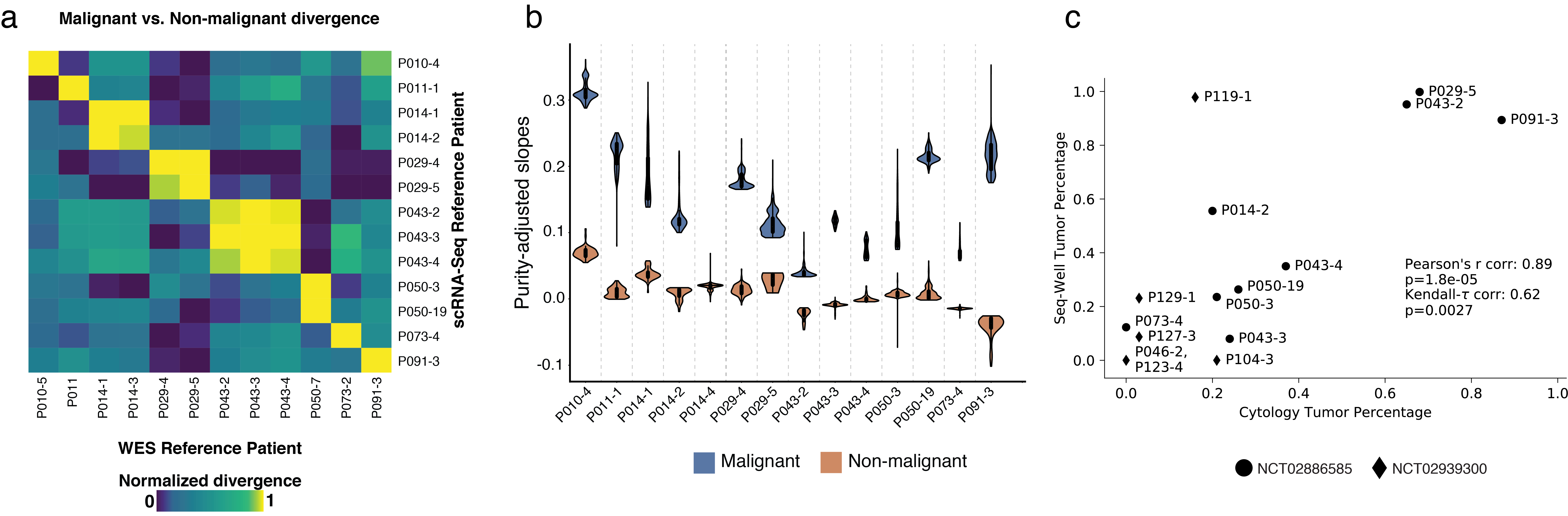


**Supplementary Fig. 3: Malignant vs. non-malignant calling by cfDNA CNV comparison.** a) The scaled difference in mean of slopes between all malignant and non-malignant cells from each sample of trial NCT02886585 is plotted as a heatmap across all combinations of time points for both single-cell RNA-Seq and cfDNA-derived reference WES data. b) The slopes for both malignant and non-malignant cells at each time point in NCT02886585 are plotted as violins. c) Tumor fraction as measured by cytology (x-axis) and detected by Seq-Well (y-axis). Kendall’s т correlation and associated p-value indicated. CSF0119-1 not included in correlation calculation as cytology for this sample was not performed simultaneous to CSF collection. P-value for Pearson’s r correlation given by Student’s t-distribution (two-sided), p-value for Kendall-τ correlation described in Methods (two-sided).


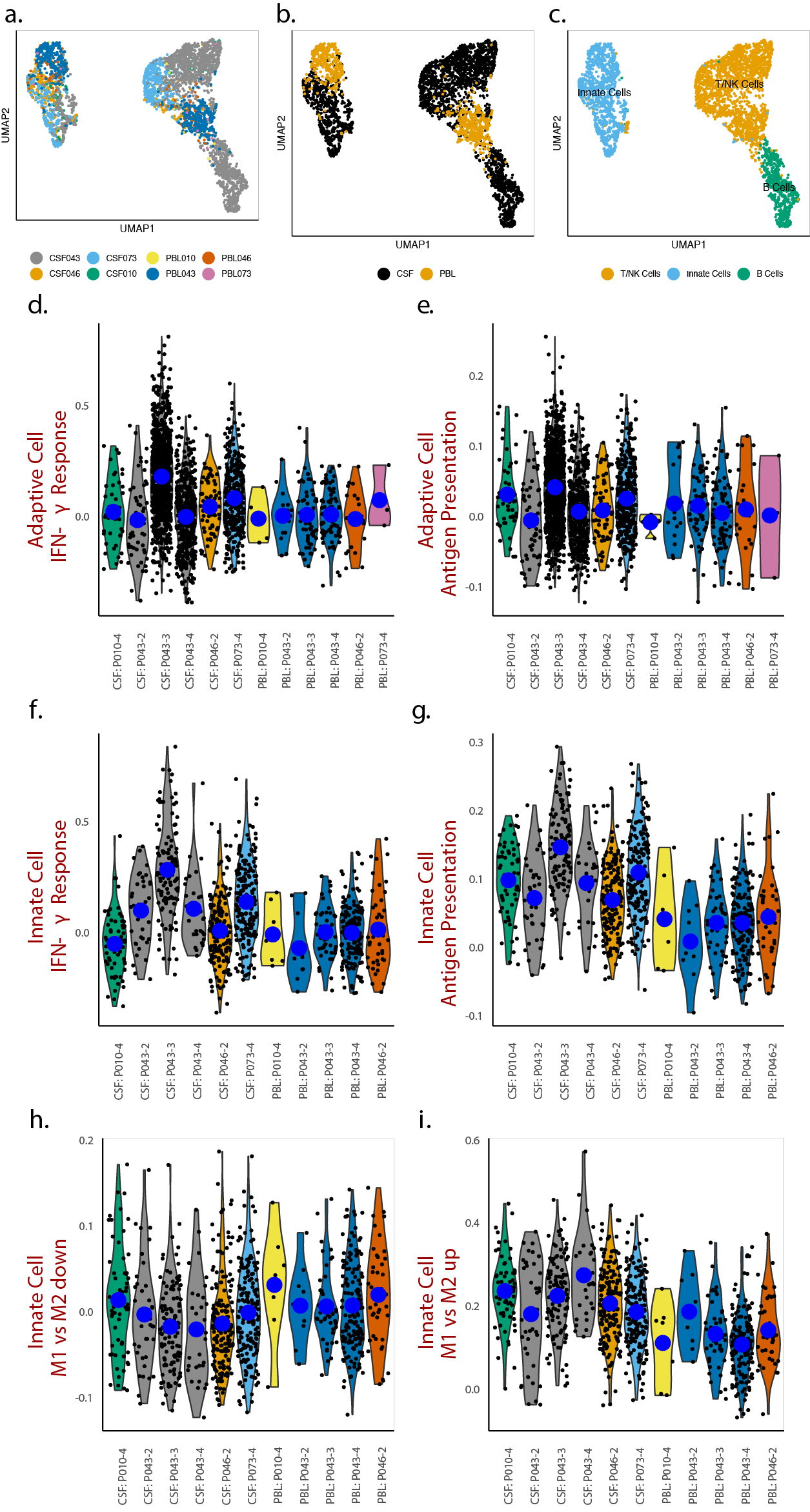


**Supplementary Fig. 4: Peripheral blood leukocytes (PBL) vs. CSF-derived single cell data at matched time points.** a,b,c) UMAP of single-cell transcriptomes across all matched samples between CSF- and PBL-derived data, colored by patient (a), location (b) and cell type (c). d-e) Violin plots of IFN-γ signaling and antigen presentation in lymphoid cells. f-g) Violin plots of IFN-γ signaling and antigen presentation in myeloid cells. h-i) Violin plots of IFN-γ M1 vs M2 Up and M1 vs M2 Down signatures in myeloid cells. P-values and effect sizes of changes between matched CSF and PBL samples, and between subsequent time points in P043, are given in Supplementary Data 6. Mean values in violins indicated by blue dots.


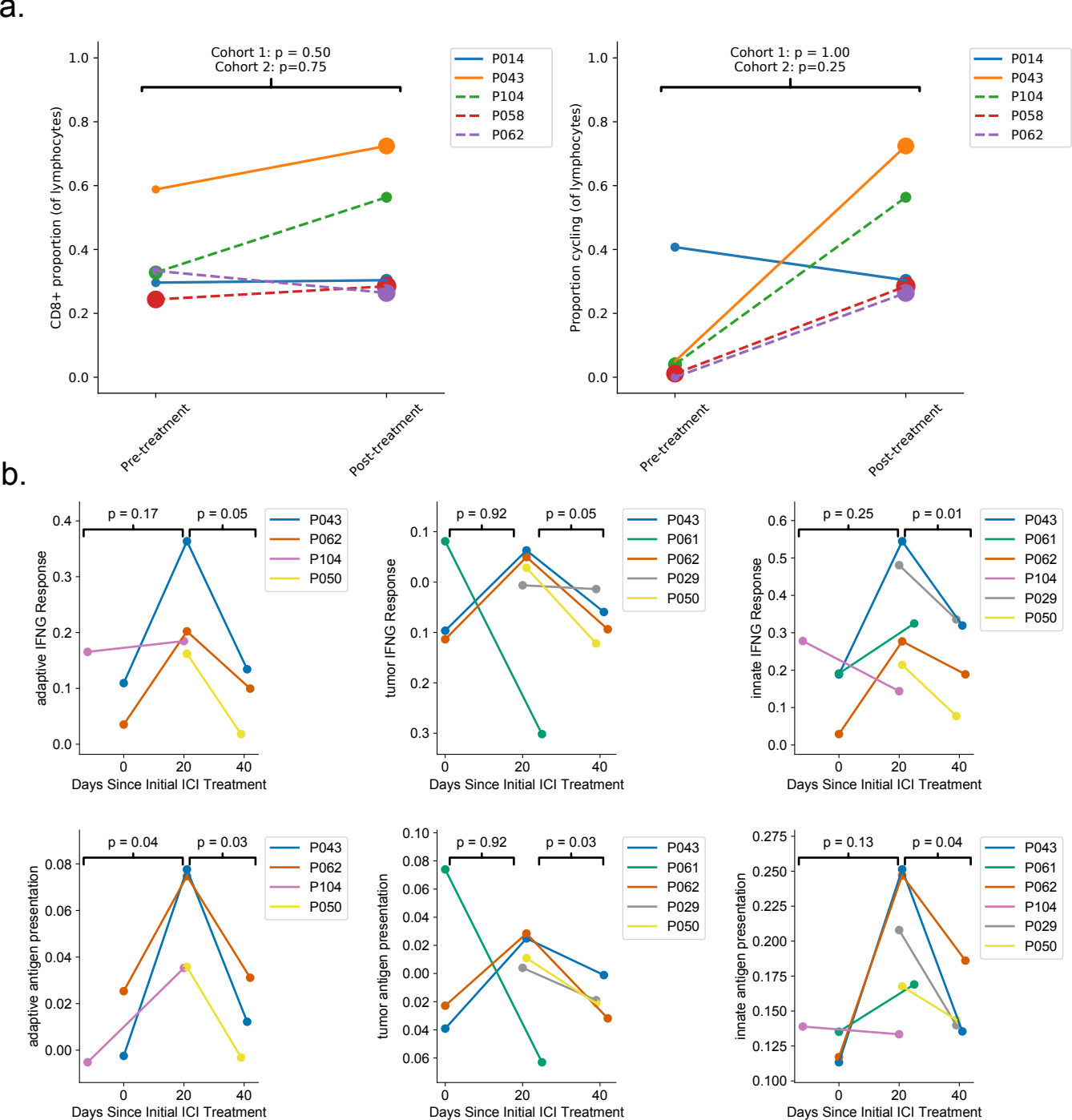


**Supplementary Fig. 5.** **Analyses of tumor and immune phenotypes restricted to longitudinally matched patient samples.** a) Comparison of pre-treatment, post-treatment cohort 1, and post-treatment cohort 2 CD8+ T cell proportion and cycling CD8+ T cell proportion restricted to longitudinally matched patient samples. b) Longitudinal analysis of tumor and immune phenotypes restricted to longitudinally matched patient samples. Indicated p-values are calculated via a dependent sample t-test.


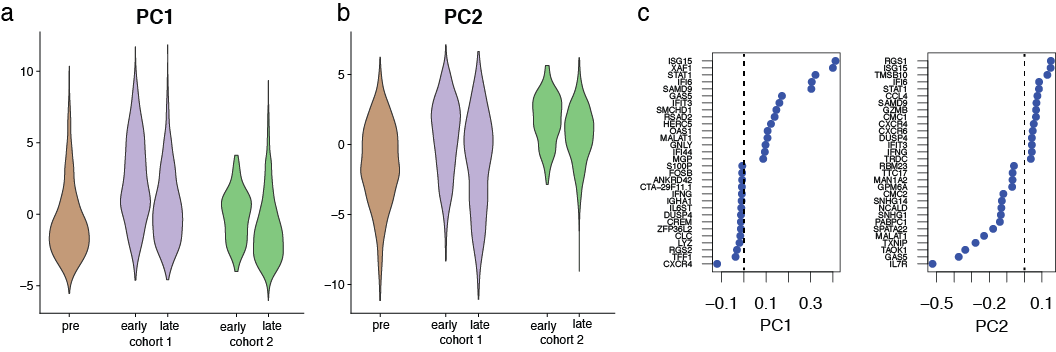


**Supplementary Fig. 6:** **Unsupervised analysis of CD8+ T cells.** a,b) Violin plots of CD8+ T cell coordinates of principal component 1 and 2, separated by pre-treatment, early (<30 days since first administration) and late (>30 days since first administration) treatment. c) The top 15 positive and negative loadings for PC1 and PC2 visualized as a dotplot.


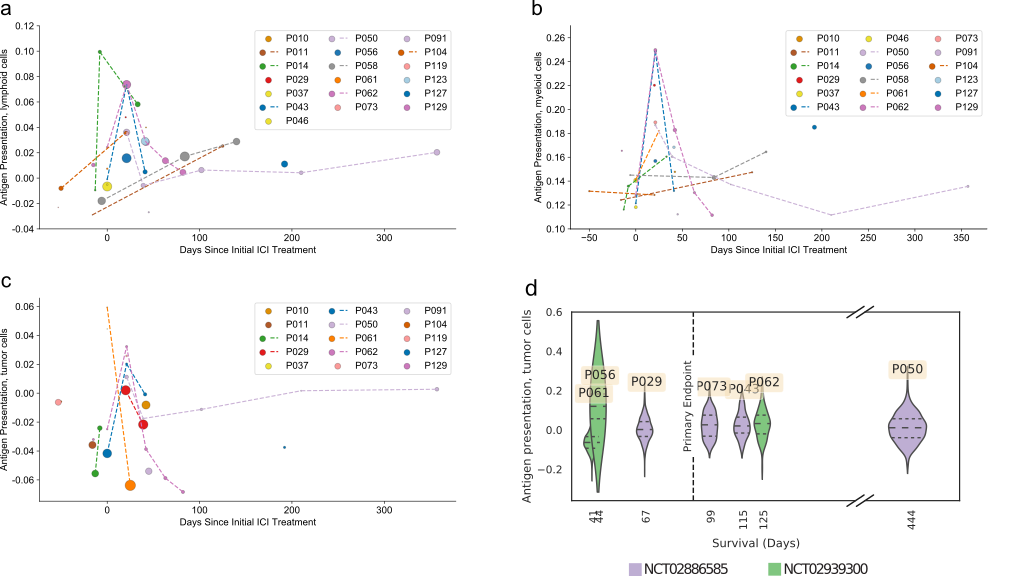


**Supplementary Fig. 7: Acute antigen presentation in CSF following intravenous ICI and relationship to survival**. Mean module score for antigen presentation within samples over time points for lymphoid (a), myeloid (b), tumor cells (c). Samples from a single patient are connected with a dashed line. The size of markers is proportional to the number of relevant cells in a sample. Points at 0 days relative to ICI administration are pre-treatment. d) Violin plots of antigen presentation for tumor cells plotted against survival (time-on-trial), for samples taken <30 days after initial ICI administration. Means and upper and lower quartiles are labeled in each violin plot.


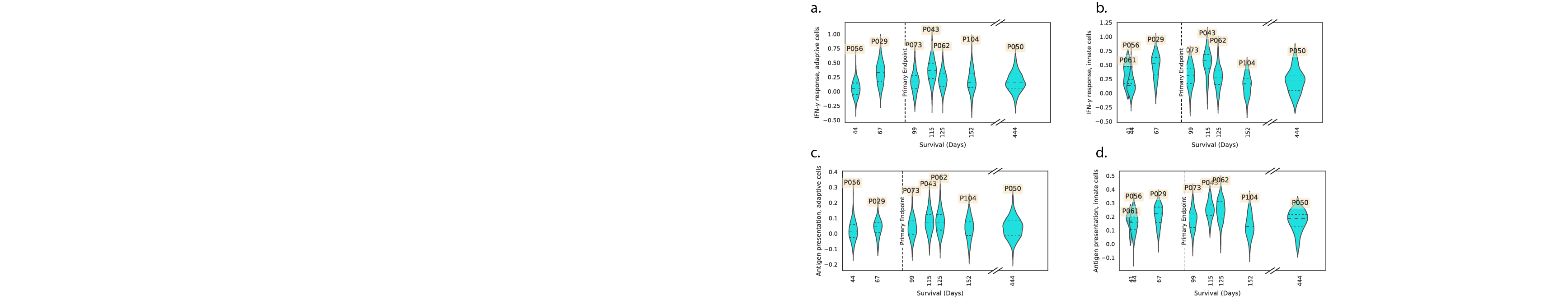


**Supplementary Fig. 8: Acute inflammatory response in immune cells and survival (time on trial)**. a-d) Violin plots of IFN-γ (a,b) and antigen presentation (c,d) for adaptive (a,c) and innate (b,d) immune cells plotted against survival (time-on-trial), for samples taken <30 days after initial ICI administration. Means and upper and lower quartiles are labeled in each violin plot.


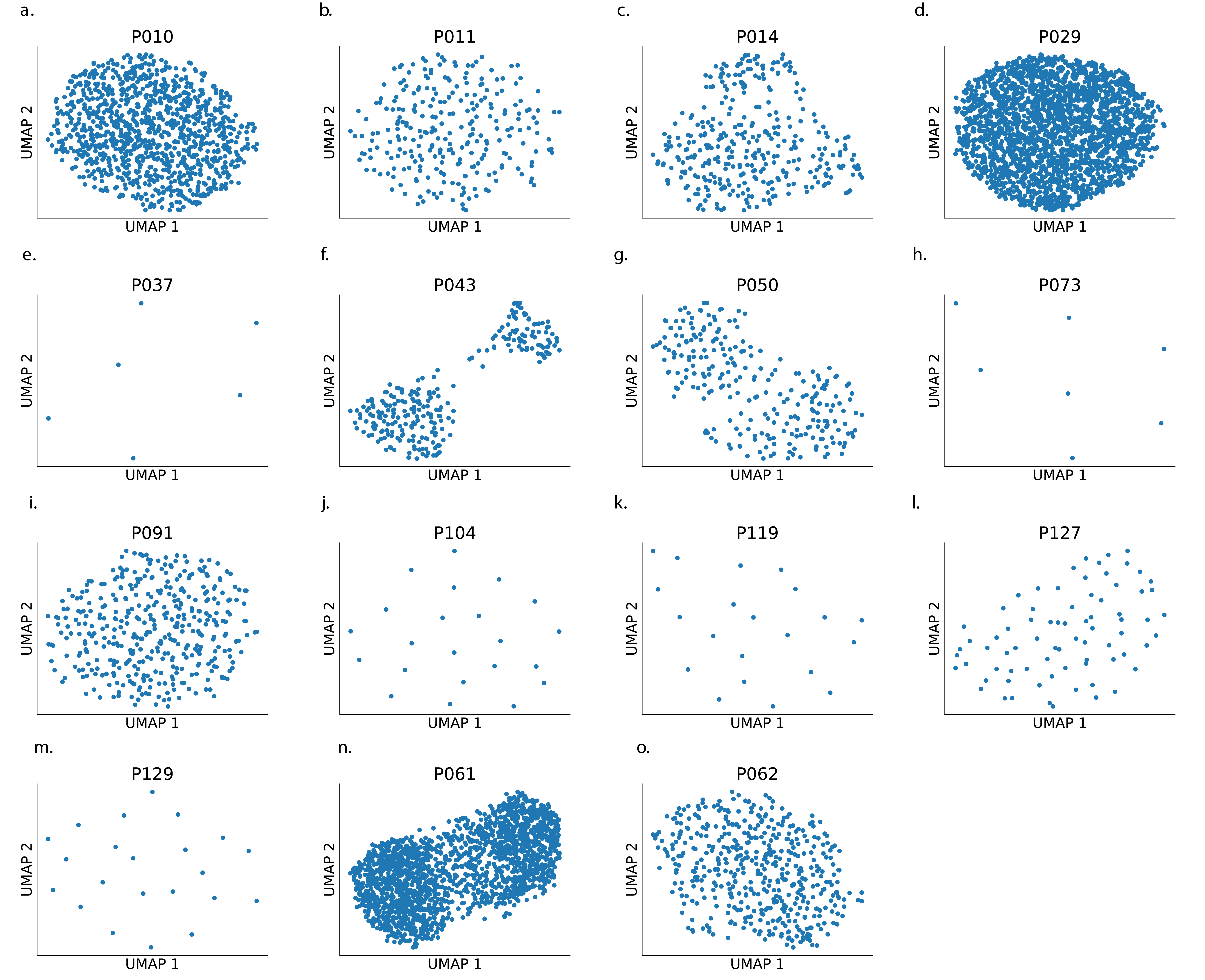


**Supplementary Fig. 9.** **UMAPs of tumor cell windowed mean expression by patient.** UMAPs calculated over rWME for 15 patients with non-zero numbers of tumor cells.


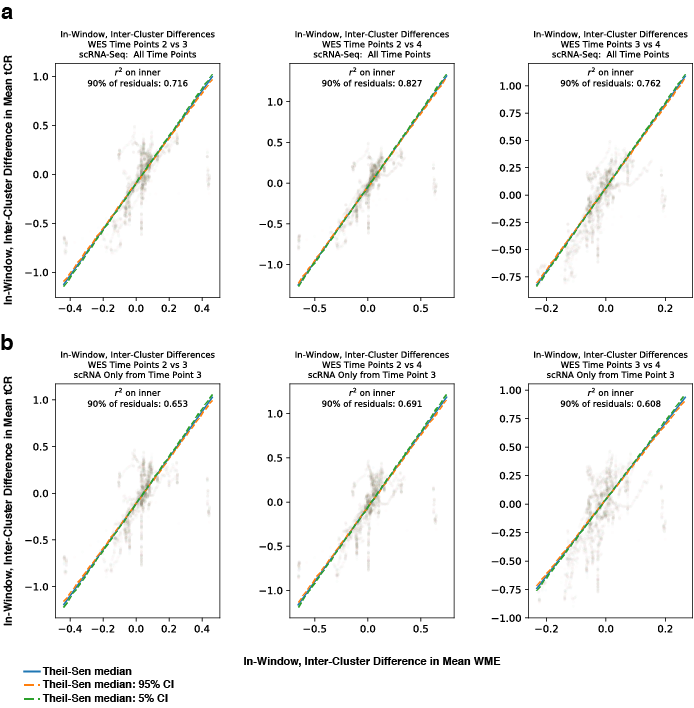


**Supplementary Fig. 10: Difference in windowed mean expression (scRNA-Seq) vs. difference in tCR (WES) at all time point combinations in P043.** a,b) Comparison of difference of expression between subclones (identified by agglomerative clustering over rWME signal of cells with complexity greater than 1000) and difference in copy number profiles at early (time point 2) and late (time point 4) time points. *x-axis*: Difference in WME, averaged within subclonal clusters, at a given gene window (mean over 200 genes contiguous along genome). *y-axis*: Difference in 200-gene-window-averaged CNV profile between CNV profiles derived from cfDNA obtained at time point 4 and time point 2. Plots in a) take cells from all time points into consideration. Plots in b) take into consideration only cells obtained at time point 3 (intermediate time point). Theil-Sen slopes indicated with 5% and 95% confidence intervals. r^2^ on inner 90% of residuals (residuals above the 95th and below the 5th percentile are ignored) given.





**Supplementary Fig. 11: Additional evidence of CNV-distinguishable subclonality in P043.** a) Heatmap of ranked windowed mean expression (rWME) ordered by chromosome and transcriptional start site for all considered tumor cells in P043, separated by agglomerative cluster identity (bottom, arrows identify peaks of mutual information between clusters). Windows are represented in transcriptomic units (i.e. units of 200 genes continuous along the genome). Plotted above is mean difference in single-cell gene expression between ascendant and descendant clusters overlaid with change in tCR between time points. WES tCR (ranks-by-window) for each of three sampled time points from CSF-derived cfDNA at top. b) Results of InferCNV performed on scRNA-Seq data from cells of P043. c) Upper row: Difference in Kendall’s τ correlations between cells’ gene expression and copy number profiles derived from cfDNA obtained at time point 4 (late) vs. time point 2 (early), distribution over all single cells in P043. Lower row: Scatter plot of cells’ IFN-γ response score and cells’ difference in Kendall’s τ correlation between cells’ gene expression and copy number profiles derived from cfDNA obtained at time point 4 (late) vs. time point 2 (early), with Theil-Sen median slopes plotted in each scatter plot (red line) with 10^th^ and 90^th^ percentile of median slopes (blue, dotted lines). d) Violin plots of antigen processing scores for cells in the ascendant and descendant subclones over time (*** p<0.001, ** p<0.01, Wilcoxon rank-sum test, Cohen’s *d* = 0.75, N = 52 for descendant, 19 for ascendant at P043-3; Cohen’s *d* = 1.15, N = 14 for descendant, 31 for ascendant at P043-4).
