# Supplementary material for: Genomic and transcriptomic correlates of immunotherapy response within the tumor microenvironment of leptomeningeal metastases
Source: Nat Commun. 2021 Oct 12;12:5955. doi: 10.1038/s41467-021-25860-5 (PMC8511044; doi:10.1038/s41467-021-25860-5)
Supplement: Supplementary file 2 — Descriptions of Additional Supplementary Files [file 41467_2021_25860_MOESM2_ESM.pdf]

Descriptions of Additional Supplementary Files

Supplementary data 1
Description: All clinical and transcriptomic summary metadata. Sample metadata includes days since initial treatment, mean transcriptomic scores for key signatures by cell compartment, and cell quality metrics. Subsequent pages detail additional clinical information, including cytology, steroid status, and Ommaya/VP shunt status.

Supplementary data 2
Description: Differential expression results between clusters, used to identify cell types, and summarized in heatmap form in Supplementary Figure 2.

Supplementary data 3
Description: Iterative cell clustering results of T cells.

Supplementary data 4
Description: Differential expression results between CD4, CD8, NK, and Cycling T cell subsets.

Supplementary data 5
Description: Gene sets used to derive key signatures throughout the manuscript.

Supplementary data 6
Description: Details of key statistical tests reported in manuscript for cerebrospinal fluid vs. peripheral blood leukocyte (CSF vs. PBL), summarized in Supplementary Figure 4.

Supplementary data 7
Description: Differential expression results between cell types found in cerebrospinal fluid vs. peripheral blood leukocyte (CSF vs. PBL).

Supplementary data 8
Description: Top genes ranked by absolute fold change in copy number ratio of CSF-derived cell-free DNA in P043 time point 4 vs. time point 2.

Supplementary data 9
Description: GSEA results distinguishing classical dendritic cells (cDCs) from plasmacytoid dendritic cells (pDCs).

Supplementary data 10
Description: Sequences of oligos used in the Seq-Well protocol.
